# Role of Acetyl-Phosphate in Activation of the Rrp2-RpoN-RpoS Pathway in *Borrelia burgdorferi*

**DOI:** 10.1371/journal.ppat.1001104

**Published:** 2010-09-16

**Authors:** Haijun Xu, Melissa J. Caimano, Tao Lin, Ming He, Justin D. Radolf, Steven J. Norris, Frank Gheradini, Alan J. Wolfe, X. Frank Yang

**Affiliations:** 1 Institute of Insect Science, Zhejiang University, Hangzhou, China; 2 Department of Microbiology and Immunology, Indiana University School of Medicine, Indianapolis, Indiana, United States of America; 3 Department of Medicine, University of Connecticut Health Center, Farmington, Connecticut, United States of America; 4 Department of Genetics and Developmental Biology, University of Connecticut Health Center, Farmington, Connecticut, United States of America; 5 Department of Pathology and Laboratory Medicine, University of Texas Medical School at Houston, Houston, Texas, United States of America; 6 National Institute of Allergy and Infectious Diseases, Rocky Mountain Laboratories, Hamilton, Montana, United States of America; 7 Department of Microbiology and Immunology, Loyola University Chicago, Stritch School of Medicine, Maywood, Illinois, United States of America; Medical College of Wisconsin, United States of America

## Abstract

*Borrelia burgdorferi*, the Lyme disease spirochete, dramatically alters its transcriptome and proteome as it cycles between the arthropod vector and mammalian host. During this enzootic cycle, a novel regulatory network, the Rrp2-RpoN-RpoS pathway (also known as the σ^54^–σ^S^ sigma factor cascade), plays a central role in modulating the differential expression of more than 10% of all *B. burgdorferi* genes, including the major virulence genes *ospA* and *ospC*. However, the mechanism(s) by which the upstream activator and response regulator Rrp2 is activated remains unclear. Here, we show that none of the histidine kinases present in the *B. burgdorferi* genome are required for the activation of Rrp2. Instead, we present biochemical and genetic evidence that supports the hypothesis that activation of the Rrp2-RpoN-RpoS pathway occurs via the small, high-energy, phosphoryl-donor acetyl phosphate (acetyl∼P), the intermediate of the Ack-Pta (acetate kinase-phosphate acetyltransferase) pathway that converts acetate to acetyl-CoA. Supplementation of the growth medium with acetate induced activation of the Rrp2-RpoN-RpoS pathway in a dose-dependent manner. Conversely, the overexpression of Pta virtually abolished acetate-induced activation of this pathway, suggesting that acetate works through acetyl∼P. Overexpression of Pta also greatly inhibited temperature and cell density-induced activation of RpoS and OspC, suggesting that these environmental cues affect the Rrp2-RpoN-RpoS pathway by influencing acetyl∼P. Finally, overexpression of Pta partially reduced infectivity of *B. burgdorferi* in mice. Taken together, these findings suggest that acetyl∼P is one of the key activating molecule for the activation of the Rrp2-RpoN-RpoS pathway and support the emerging concept that acetyl∼P can serve as a global signal in bacterial pathogenesis.

## Author Summary

*Borrelia burgdorferi*, the causative agent of Lyme disease, is maintained in nature in a complex enzootic cycle involving *Ixodes* ticks and mammals. A novel regulatory network, the Rrp2-RpoN-RpoS pathway, which governs differential expression of numerous genes of *B. burgdorferi*, is essential for this complex life cycle. In this study, we provide evidence showing that the activation of the Rrp2-RpoN-RpoS pathway is modulated, not by the predicted histidine kinase for Rrp2, but rather by acetyl phosphate (acetyl∼P), the intermediate of the Ack-Pta (acetate kinase-phosphate acetyltransferase) metabolic pathway. Based on our findings, we propose that during the enzootic cycle of *B. burgdorferi*, changes in environmental cues and nutrient conditions lead to an increase in the intracellular acetyl∼P pool in *B. burgdorferi*, which in turn modulates the activation of the Rrp2-RpoN-RpoS pathway.

## Introduction

The enzootic life-cycle of *Borrrelia burgdorferi* is complex and typically involves transmission between an arthropod vector (*Ixodes* ticks) and a mammalian host (e.g., *Peromyscus* rodents) [1]. Accumulated evidence have shown that the alternative sigma factor RpoS plays a central role in this complex natural cycle of *B. burgdorferi* [2]–[8]. RpoS functions as a global regulator and governs differential expression of more than 10% of all *B. burgdorferi* genes, including the two major virulence genes *ospA* and *ospC* [9]–[13]. One unique feature about *rpoS* of *B. burgdorferi* is that its expression is directly controlled by the alternative second sigma factor RpoN (σ^54^) at a −24/−12 σ^54^-type promoter. Mutation within this promoter region or inactivation of *rpoN* that encodes the second alternative sigma factor RpoN (σ^54^) abolishes expression of *rpoS* and RpoS-dependent genes such as *ospC* [6], [8], [14]. This RpoN-dependent transcriptional activation appears to play a major role in modulating RpoS level in *B. burgdorferi* [3], [5]–[8], [14], [15]. In addition, a small RNA *dsrA* also has been shown to be involved in post-transcriptional regulation of RpoS [7].

RpoN(σ^54^)-dependent activation of transcription requires a highly conserved transcriptional activator, the so-called enhancer-binding proteins (EBPs) [16]. *B. burgdorferi* has a single EBP, Rrp2, a homolog of NtrC family [17], [18]. Members of NtrC family contain three putative functional domains: an N-terminal response regulator receiver domain, a central RpoN-activation domain, and a C-terminal helix-turn-helix (HTH) DNA-binding domain [19]. The central domain becomes activated upon phosphorylation at a conserved aspartic acid residue (corresponding to D52 in Rrp2) within the N-terminal receiver domain. The activated central domain then contacts the Eσ^54^-holoenzyme through DNA looping, hydrolyzes ATP, and promotes open promoter complex formation for transcriptional initiation. Although direct biochemical evidence remains lacking, genetic data indicates that Rrp2 is the activator for the σ^54^–σ^S^ cascade of *B. burgdorferi*. First, a single point mutation of glycine (G) residue 239 to cysteine (C) within one of the ATP-binding motifs in the central activation domain of Rrp2 abolishes expression of *rpoS* and RpoS-dependent genes [4], [18], [20]. Second, when a *rpoS* promoter*-cat* reporter and an inducible *rrp2* gene were cloned into a surrogate *E. coli* system, the reporter was activated only upon induction of *rrp2* [6]. Thus, Rrp2, RpoN, and RpoS appear to constitute a Rrp2-RpoN-RpoS pathway. Consistent with this notion, recent microarray analyses reveal that genes influenced by Rrp2, RpoN, or RpoS largely overlap [2]–[4], [20].

Given the importance of the Rrp2-RpoN-RpoS pathway to the infectious cycle of *B. burgdorferi* [3]–[5], [20], it is striking how little we know about the upstream event(s) that lead to its activation. Since Rrp2 is the upstream activator for the pathway, an understanding of the activation of Rrp2 is key to understand the mechanism of activation of this pathway. It is postulated that activation of Rrp2 is through a phosphorylation event by a cognate histidine kinase [21]–[23]. Because of the co-localization of *rrp2* and *hk2* in the genome (15) and because of the ability of Hk2 to phosphorylate Rrp2 *in vitro* [6], Hk2 is predicted to be the cognate histidine kinase for Rrp2. A recent study by Burtnick *et al*. [6], however, showed that an *hk2* mutant remains capable of activating Rrp2 under *in vitro* cultivation conditions, indicating that the molecular mechanism activating the Rrp2-RpoN-RpoS pathway is more complex than previously envisioned. In addition, the contribution of Hk2 during the infectious cycle of *B. burgdorferi* remains unknown because the previous *hk2* mutant lost an important endogenous plasmid (lp36) for mammalian infection [6].

Response regulators can be activated by factors other than their cognate histidine kinases. The best studied mechanisms are phosphorylation by non-cognate histidine kinases (a phenomenon called “cross-talk”) [24]–[28] and phosphorylation by small molecular weight high-energy donors, such as acetyl phosphate (acetyl∼P) or carbamoyl phosphate (carbamoyl∼P) [29]–[31]. While cross-talk appears to be quite rare (48), emerging evidence indicates that acetyl∼P can function *in vivo* as a global signal by donating its phosphoryl group to certain response regulators [32], [33]. *B. burgdorferi* possesses four predicted histidine kinases (Hk1, Hk2, CheA1, and CheA2) [17], [34] as well as pathways for the synthesis and degradation of both acetyl∼P and carbamoyl∼P [17]. Burtnick *et al.* [6] proposed that Hk2-independent activation of Rrp2 could be activated by receiving a phosphoryl group from a non-cognate histidine kinase or a small phosphorylated compound. However, this hypothesis has not been tested experimentally. In this study, we generated an *hk2* mutant suitable for *in vivo* study and showed that Hk2 was not required for the activation of the Rrp2-RpoN-RpoS pathway under *in vitro* growth conditions or during murine infection. We further showed that cross-talk among two-component systems is not likely to account for Rrp2 activation. Rather, the results obtained support the hypothesis that acetyl∼P functions as an important phosphoryl donor for Rrp2, making this small molecule a key modulator of the activation of the Rrp2-RpoN-RpoS pathway in *B. burgdorferi*.

## Results

### Hk2 is not required for the activation of Rrp2-RpoN-RpoS pathway in mammalian host-adapted spirochetes or during murine infection

**Figure 1:**
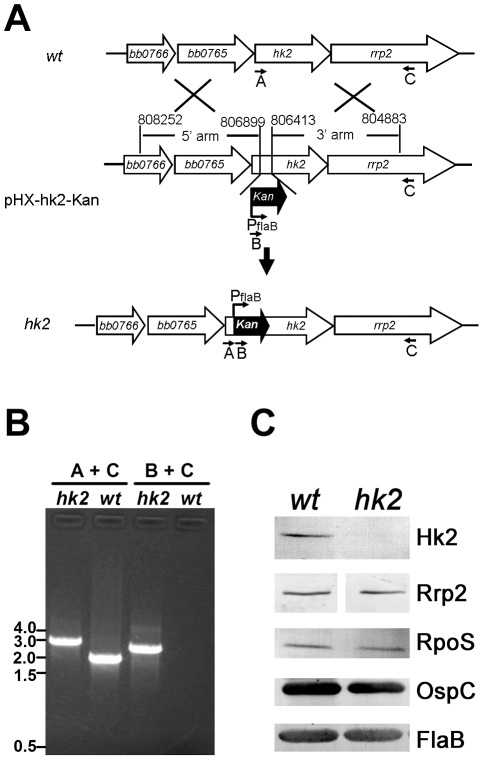
Construction and characterization of the *hk2* mutant. (**A**) Strategy for insertional inactivation of *hk2*. *wt*, genomic structure of *hk2* and the surrounding region in wild-type *B. burgdorferi*. pHX-hk2-Kan, the suicide vector used for generating the *hk2* mutant. Only the relevant portion of the plasmid is shown. *hk2*, the diagram showing the genomic structure of the *hk2* mutant. Small labeled arrows denote positions of oligonucleotide primers used for PCR analyses. (**B**) Confirmation of the *hk2* mutant by PCR analyses. Letter combinations denote primer pairs used for PCR. kb: kilobase DNA ladder, *wt*, wild type strain; *hk*2, *hk2* mutant. (**C**) Immunoblot analyses of the *hk2* mutant. Cultures were grown at 35°C to late logarithmic phase (5×10^7^ spirochetes/ml) and subjected to immunoblotting with monoclonal antibodies against Hk2, Rrp2, RpoS, OspC or FlaB (loading control).

To study the mechanism of activation of the Rrp2-RpoN-RpoS pathway, we focused on the upstream activator Rrp2, a putative response regulator. Burtnick *et al*. [6] recently reported that inactivation of *hk2*, which encodes the putative cognate histidine kinase for Rrp2, did not affect activation of the Rrp2-RpoN-RpoS pathway when spirochetes were cultivated *in vitro*. However, this *hk2* mutant was not phenotypically characterized *in vivo* [6]. Thus, we sought to generate an *hk2* mutant suitable for *in vivo* study. A suicide vector harboring a disrupted *hk2* region was transformed into the infectious *B. burgdorferi* strain B31-A3 (**Fig. 1A**) [35]. Disruption of *hk2* in the transformants was confirmed by PCR (**Fig. 1B**) and the absence of Hk2 expression was verified by immunoblot analyses (**Fig. 1C**). Of note, inactivation of *hk2* by the Kan^R^ cassette did not substantially affect expression of the protein encoded by the downstream gene, *rrp2* (**Fig. 1C**). Three transformed clones were further subjected to plasmid profile analyses (data not shown). Two clones had a plasmid profile identical to that of parental wild-type B31-A3; one of these was designated *hk2* and chosen for further study (**Table 1**).

**Table 1:** Strain description.

Spirochete	Description	Plasmid missing	Source
B31-A3	A low–passage and virulent strain, derived from B31-MI	cp9	[35]
B31 13A	A non-infectious clone, derived from B31	lp25, lp56	[59]
B31 5A18	An infectious clone, derived from B31-MI	lp28-4, lp56	[63]
BbAH130	An infectious clone, derived from *Bb* strain 297		[9]
*rrp2*	Same as BbAH130, except *rrp2* was replaced with *rrp2(G239C)*		[18]
*rpoS*	Same as Bb297, except *rpoS* was disrupted with a *ermC* selection marker		[8]
*hk2*	B31-A3 with the *hk2* gene (*bb0764*) disrupted with a kanamycin selection marker		This study
*hk1*	AH130 with the *hk1* gene (*bb0420*) disrupted with a *ermC* selection marker		This study
*hk1 hk2*	An *hk1 hk2* double mutant in AH130, generated by transforming the *hk2* suicide vector into the *hk1* mutant		This study
*cheA1*	A *cheA1* (*bb0567*) mutant in B31-A	lp28-4, lp56	[44]
*cheA2*	A *cheA2* (*bb0669*) mutant in B31-A	lp25, lp56	[44]
*arcA*	An *arc* (*bb0841*) mutant, generated by transposon-mediated mutagenesis in B31 5A18	lp25, lp56	This study
13A/Rrp2-N	B31 13A carrying a shuttle vector that overexpresses Rrp2-N	lp25, lp56	This study
13A/D52A	B31 13A carrying a shuttle vector that overexpresses Rrp2-N(D52A)	lp25, lp56	This study
13A/D52E	B31 13A carrying a shuttle vector that overexpresses Rrp2-N(D52E)	lp25, lp56	This study
13A/vector	B31 13A carrying a shuttle vector alone	lp25, lp56	This study
13A/Pta	B31 13A carrying a shuttle vector that overexpresses Pta	lp25, lp56	This study
A3/Rrp2-N	B31-A3 carrying a shuttle vector that overexpresses Rrp2-N	cp9	This study
A3/D52A	B31-A3 carrying a shuttle vector that overexpresses Rrp2-N(D52A)	cp9	This study
A3/Pta	B31-A3 carrying a shuttle vector that overexpresses Pta	cp9	This study

Under *in vitro* growth conditions, a combination of elevated temperature and increased cell density activates the Rrp2-RpoN-RpoS pathway, leading to the production of RpoS and RpoS-controlled proteins such as OspC [2], [5], [6], [8], [18], [36]–[39]. To determine if Hk2 affects temperature and cell density-dependent activation of the Rrp2-RpoN-RpoS pathway, wild-type *B. burgdorferi* and isogenic *hk2* mutant spirochetes were cultivated at elevated temperature (35°C) and harvested at the late-exponential stage of growth (5×10^7^ spirochetes per ml), conditions under which the Rrp2-RpoN-RpoS pathway is known to be activated. The *hk2* mutant and its parental strain expressed similar levels of RpoS and OspC (**Fig. 1C**). Under “non-inducing” conditions (i.e., low cell density or lower culture temperature), neither the *hk2* mutant nor the parent strain expressed OspC (data not shown). Thus, consistent with studies by Burtnick *et al*. [6], the Rrp2-RpoN-RpoS pathway can be activated *in vitro* in an Hk2-independent manner.

**Figure 2:**
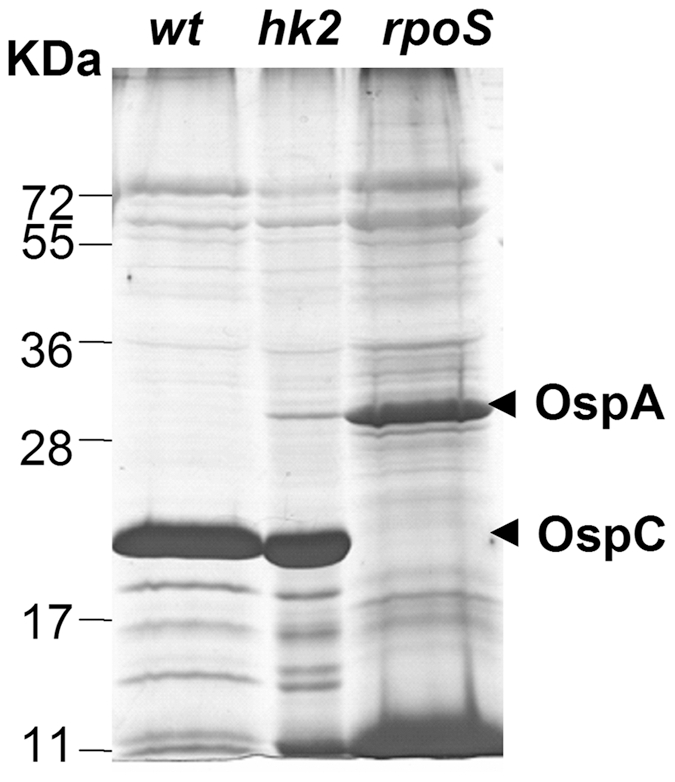
*hk2* mutant spirochetes cultivated in DMCs are capable of activating the Rrp2-RpoN-RpoS pathway. Wild-type (*wt*), *hk2* mutant (*hk2*), or *rpoS* mutant (*rpoS*) spirochetes were cultivated in DMCs, after which whole cell lysates were separated by SDS-PAGE and visualized with silver stain. The bands corresponding to OspA and OspC are indicated by arrowheads on the right. Molecular mass markers in kilodaltons are shown on the left.

*In vitro* growth conditions only partially mimic the *B. burgdorferi* gene expression patterns observed during tick feeding and mammalian infection. For example, spirochetes grown under elevated temperature and high cell density conditions upregulate *ospC* but do not downregulate *ospA* [2], [40]–[42]. Therefore, we next examined the phenotype of the *hk2* mutant grown in mammalian host-adapted conditions by cultivating spirochetes in dialysis membrane chambers (DMCs) implanted in the peritoneal cavities of rats [2], [40]–[42]. As shown in **Fig. 2**, wild-type spirochetes cultivated in DMCs produced large amounts of OspC and undetectable amounts of OspA. An *rpoS* mutant exhibited the opposite phenotype, as previously reported [41]. In contrast, the DMC-cultivated *hk2* mutant behaved much like its wild-type parent, indicating that Hk2 was not required for Rrp2 activation within this mammalian host environment.

To further determine whether Hk2 is required for murine infection, groups of C3H/HeN mice were inoculated intradermally with various doses of either wild-type *B. burgdorferi* B31-A3 or its isogenic *hk2* mutant. As shown in **Table 2**, the infectivity of the *hk2* mutant was similar to that of the parental strain. This result suggests that unlike Rrp2, RpoN and RpoS [3]–[5], [20], Hk2 was not required for infection of mice by *B. burgdorferi*.

**Table 2:** Mouse infectivity of the *hk2* mutant.

Strains	No. of mouse tissues culture positive/total No. of tissues tested				No. of mice infected/total No. of mice
	Skin	Heart	Joint	Bladder	
B31-A3					
10^5^	5/5	5/5	5/5	5/5	5/5
10^3^	9/10	9/10	9/10	9/10	9/10
*hk2* mutant					
10^5^	5/5	5/5	5/5	5/5	5/5
10^3^	7/10	7/10	8/10	8/10	8/10

### Other histidine kinases are not involved in Rrp2 activation

**Figure 3:**
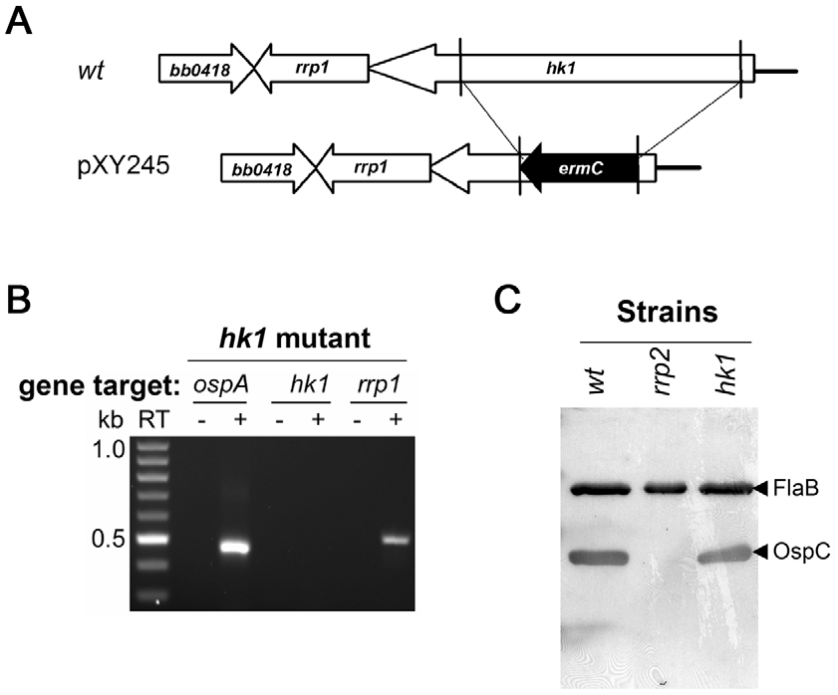
The *hk1* mutant remains capable of activating the Rrp2-RpoN-RpoS pathway. (**A**) Strategy for insertional inactivation of *hk1*. *wt*, genomic structure of *hk1* in wild-type *B. burgdorferi*. pXY245, the suicide vector used for generating the *hk1* mutant. Only the relevant portion of the plasmid is shown. (**B**) Confirmation of the *hk1* mutant by RT-PCR analyses. RT-PCR was performed using primers specific for *ospA*, *hk1*, or *rrp1* (labeled on the top). kb: the kilobase DNA ladder. RT indicates the absence (-) or presence (+) of reverse transcriptase in the reaction. (**C**) Production of OspC by the *hk1* mutant. Various strains of spirochetes (labeled on the top) were grown at 35°C and harvested in the late logarithmic phase (5×10^7^ spirochetes/ml) and subjected to immunoblot analysis using a mixture of monoclonal antibodies specific for OspC and FlaB, respectively. A strain harboring a G239C point mutation within Rrp2 [18], serves as a negative control for OspC expression. The bands corresponding to OspC or FlaB are indicated by the arrowhead on the right.

The results described above indicate that Rrp2 could be activated by an Hk2-independent mechanism. To test the possibility that cross-talk may contribute to Rrp2 activation, we assessed the involvement of the other three *B. burgdorferi* histidine kinases identified to date [17]. We first constructed an *hk1* mutant (*hk1*) in *B. burgdorferi* 297 using a strategy similar to that described for generating the *hk2* mutant (**Fig. 3A**). The resulting mutant was verified using RT-PCR to test for the absence of *hk1* expression and the lack of polarity on the downstream gene *rrp1* (**Fig. 3B**). Spirochetes were cultivated at elevated temperature and harvested at the late-exponential stage of growth. Unlike the *rrp2(G239C)* mutant, which failed to express OspC, the *hk1* mutant produced levels of OspC that were comparable to those of its wild-type parent, indicating that Hk1 is dispensable for Rrp2 activation (**Fig. 3C**).

**Figure 4:**
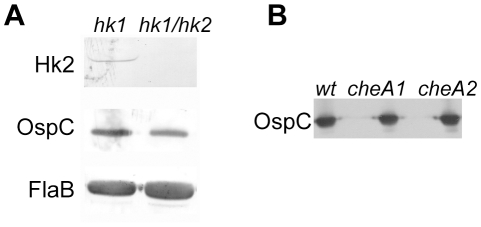
The *hk1 hk2* double mutant and the *cheA1* or *cheA2* mutant have normal level of Rrp2 activation. Various strains of spirochetes (labeled on the top) were grown at 35°C, harvested in the late logarithmic phase (5×10^7^ spirochetes/ml), and subjected to immunoblot analysis using monoclonal antibodies specific against Hk2, OspC or FlaB as indicated.

It remained possible that Hk1 and Hk2 are involved in Rrp2 activation but that they may compensate for each other in a single knockout mutant. To rule out this possibility, we generated an *hk1 hk2* double mutant in *B. burgdorferi* 297 by transforming the *hk1* mutant with the suicide vector used for generating the *hk2* mutant. Immunoblot analysis of the double mutant confirmed the absence of Hk2 in the *hk1 hk2* mutant, and, more importantly, demonstrated that temperature and cell density-induced expression of OspC was unaffected despite the loss of both histidine kinases (**Fig. 4A**). These results indicate that during *in vitro* growth, Hk1 is not responsible for Rrp2 activation in the absence of Hk2.

In addition to Hk1 and Hk2, *B. burgdorferi* expresses two other histidine kinases, CheA1 and CheA2, both of which are involved in chemotaxis [43], [44]. To determine whether CheA1 or CheA2 participate in Rrp2 activation, we examined the ability of *cheA1* and *cheA2* mutants to produce OspC. As shown in **Fig. 4B**, both *cheA* mutants expressed normal levels of OspC, indicating that neither CheA1 nor CheA2 is required for Rrp2 activation under *in vitro* growth conditions.

### Rrp2 activation requires the conserved phosphorylation site D52

**Figure 5:**
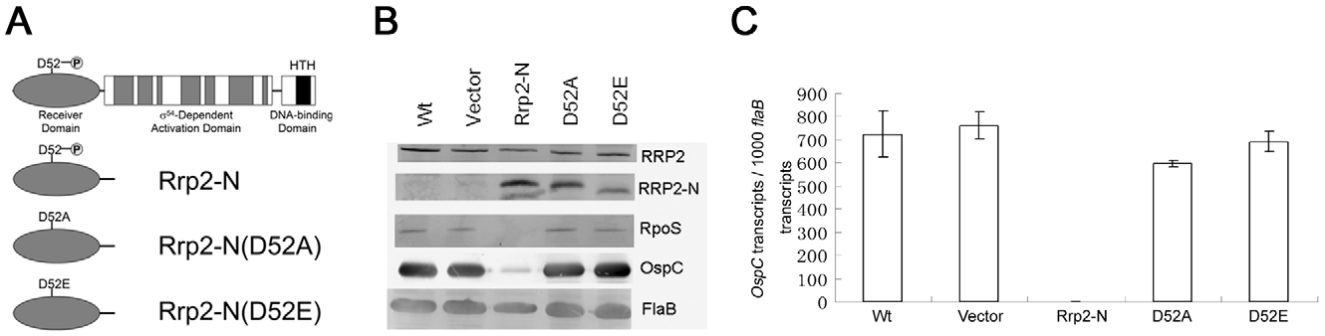
Influence of overexpression of wild-type or mutated version of the Rrp2 N-terminal receiver domain on Rrp2 activation. (**A**) Schematic diagram of predicted Rrp2 domain structure and various versions of overexpressed N-terminal receiver domains. D52 is the putative phosphorylation site. (**B**) Immunoblot of wild-type strain (lane 1), the strain carrying the shuttle vector only (lane 2), the strain with overexpression of Rrp2-N (lane 3), the strain with overexpression of Rrp2-N(D52A) (lane 4), and the strain with overexpression of Rrp2-N(D52E) (lane 5). Cultures were grown to late logarithmic phase at 35°C. Pooled antibodies/antisera against Rrp2, FlaB, and OspC were used. Bands corresponding to each protein were labeled on the right. (**C**) qRT-PCR analysis of *ospC* expression in various strains shown in (**B**). Levels of *ospC* transcript were normalized per 1000 copies of *flaB* in each sample.

As a putative two-component response regulator, it is predicted that Rrp2 becomes activated upon phosphorylation of a conserved aspartate residue (D52) located within its N-terminal receiver domain [6], [18] (**Fig. 5A**). Since deletion of each histidine kinase gene exerted no effect on the activation of the Rrp2-RpoN-RpoS pathway, we asked whether Rrp2 activation actually requires phosphorylation. Repeated attempts to replace the wild-type *rrp2* with a mutated allele containing a D52A mutation were unsuccessful. As an alternative strategy, we reasoned that, if phosphorylation is important for Rrp2 activation, overexpression of a wild-type N-terminal Rrp2 fragment (Rrp2-N) (phosphorylatable but not active) would interfere with phosphorylation of endogenous full-length Rrp2 and therefore affect activation of the Rrp2-RpoN-RpoS pathway. Conversely, overexpression of a non-phosphorylatable mutant version of the Rrp2 N-terminus should have no effect. Accordingly, we constructed a series of shuttle vectors that carried the wild-type allele rrp2-N or the mutant alleles *rrp2-N(D52A)* or *rrp2-N(D52E)* under control of the constitutive *flaB* promoter (**Fig. 5A**). Each constructed vector then was transformed into a non-infectious but highly transformable strain, B31 13A. The resulting transformants were verified by immunoblot analysis showing that each produced native full-length Rrp2 and the overexpressed Rrp2-N fragment (**Fig. 5B**). We then evaluated the ability of these transformants to express OspC. Overexpression of wild-type Rrp2-N almost completely abolished expression of *ospC* (**Fig.** **5B** and **5C**). These results were consistent with the expectation that the Rrp2-N fragment can successfully compete with native full-length Rrp2 for phosphorylation and, thus, interfere with Rrp2 and RpoN (σ^54^)-dependent transcription of *rpoS* [14], [15]. In contrast, cells expressing non-phosphorylatable Rrp2-N(D52A) or Rrp2-N(D52E) behaved like the vector control (**Fig. 5B** and **5C**), as would be expected if Rrp2 activation requires phosphorylation of D52.

Given that the Rrp2-RpoN-RpoS pathway is essential for mammalian infection, we hypothesized that overexpression of Rrp2-N, but not Rrp2-N(D52A) would affect the spirochete's ability to infect mice. To test this hypothesis, we re-transformed the corresponding shuttle vectors into the infectious strain B31-A3. Positive transformants that had endogenous plasmid profiles identical to that of B31-A3 were then needle-inoculated into groups of C3H/HeN mice. As shown in **Table 3**, although the strain overexpressing wild-type Rrp2-N was capable of infecting mice with a high dose of inoculation (1×10^5^ spirochetes per mouse), its infectivity was greatly reduced; only 1 out of 5 mice was infected at the dose of 1×10^3^ spirochetes (**Table 3**). In contrast, overexpression of Rrp2-N(D52A) exerted no such effect. Thus, overexpression of Rrp2-N impaired the activation of the Rrp2-RpoN-RpoS pathway both *in vitro* and *in vivo*, further supporting the hypothesis that phosphorylation of Rrp2 is likely required for the activation of the Rrp2-RpoN-RpoS pathway.

**Table 3:** Mouse infectivity of *Borrelia burgdorferi* with overproduction of Rrp2-N or Pta.

Strain	Ear*	No. of cultures positive/total No.				No. of mice positive/total No. of mice
		Skin	Joint	Heart	All sites	
B31-A3						
10^5^	3/3	3/3	3/3	3/3	9/9	3/3
10^3^	9/10	9/10	9/10	9/10	27/30	9/10
A3/vRrp2-N						
10^5^	2/3	2/3	2/3	2/3	6/9	2/3
10^3^	0/5	1/5	1/5	1/5	3/15	1/5
A3/vRrp2-N^D52A^						
10^3^	5/5	5/5	5/5	5/5	15/15	5/5
A3/vPta						
10^3^	4/8	4/8	3/8	4/8	11/24	4/8

*Ear punch biopsies were examined at day 10 and other tissues were examined at day 20 post inoculation.

### Carbamoyl phosphate does not contribute to Rrp2 activation

**Figure 6:**
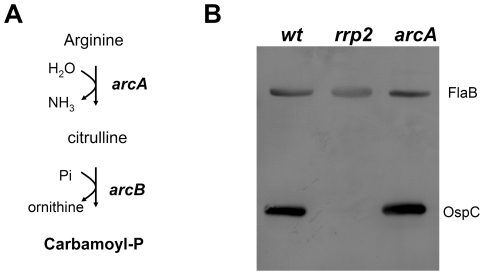
Inactivation of the carbamoyl-P biosynthesis pathway does not affect Rrp2 activation. (**A**) Diagram of the arginine fermentation pathway in *B. burgdorferi*. The *arcA (bb0841)* and *arcB (bb0842)* genes are predicted to encode arginine deaminase and ornithine carbamoyltransferase, respectively. (**B**) Immunoblot analysis of whole cell lysates of wild-type (*wt*), the *rrp2* mutant [*rrp2(G239C)*], and the *arcA* mutant (*arcA*) with a mixture of antibodies against OspC and FlaB. Spirochetes were cultured at 35°C and harvested at late logarithmic growth. The bands corresponding to FlaB and OspC are indicated on the right.

Since Rrp2 activation appears to require D52, but not the *B. burgdorferi* histidine kinases, we reasoned that small metabolic intermediates (e.g., carbamoyl∼P or acetyl∼P) might be responsible for phosphorylation of D52. The *B. burgdorferi* genome is predicted to encode a single pathway that can produce carbamoyl-P, the so-called arginine fermentation or ArcA-ArcB pathway, in which the enzyme arginine deaminase (ArcA) converts arginine to citrulline, which is then converted to ornithine and carbamoyl∼P by the enzyme ornithine carbamoyltransferase (ArcB) (**Fig. 6A**). To assess the ability of carbamoyl∼P to influence Rrp2 activation, we used transposon mutagenesis to construct an *arcA* (*bb0841*) mutant (see Materials and Methods). The *arcA* mutant had no growth defect *in vitro* (data not shown) and produced levels of OspC similar to those of the wild-type parent strain (**Fig. 6B**). Moreover, wild-type spirochetes cultivated in growth medium supplemented with an excess of arginine or ornithine showed no change in OspC expression (data not shown). Collectively, these results argue that carbamoyl∼P does not donate its phosphoryl group to activate Rrp2, at least under *in vitro* cultivation conditions.

### Acetate induces activation of the Rrp2-RpoN-RpoS pathway

**Figure 7:**
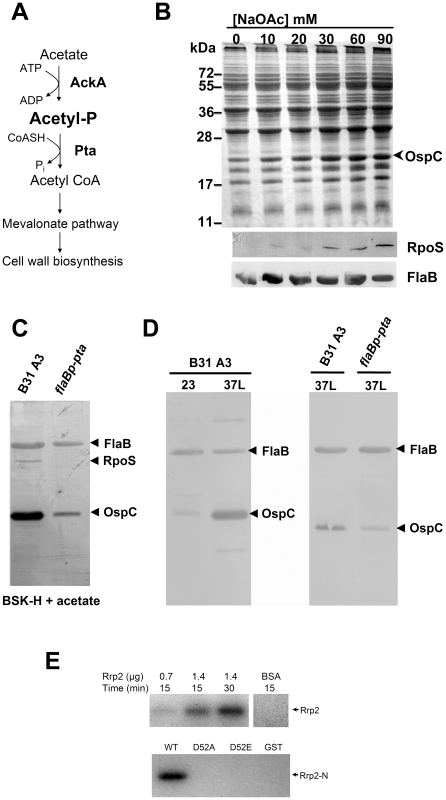
Acetyl∼P plays an important role in Rrp2 activation under *in vitro* cultivation conditions. (**A**) Diagram of the ACK-PTA pathway in *B. burgdorferi*. *ack* (*bb0622*) encodes acetate kinase (Ack), which converts acetate to the intermediate acetyl∼P, while *pta (bb0589)* encodes phosphate acetyltransferase (Pta), which synthesizes acetyl-CoA from acetyl∼P and CoASH [17]. In *B. burgdorferi*, the Ack-Pta pathway appears to be the sole pathway for biosynthesis of acetyl-CoA, a molecule required for cell membrane biosynthesis (see Results and Discussion for details). (**B**) Acetate induces activation of the Rrp2-RpoN-RpoS pathway. Wild-type *B. burgdorferi* strain B31-A3 was cultivated in the BSK-H medium supplemented with 0–90 mM NaOAc with a final media pH value of 7.0. Cells were harvested at the early-logarithmic phase (5×10^6^ spirochetes/ml). Cell lysates were subjected to SDS-PAGE (top panel) or immunoblot (bottom panels) analysis. The bands corresponding to OspC, RpoS and FlaB were labeled on the right. (**C**) Overexpression of Pta reduces acetate-induced Rrp2 activation. Wild-type *B. burgdorferi* strain B31 13A (-) or the strain carrying *flaBp-pta* (+) were cultivated in the BSK-H medium supplemented with 15 mM NaOAc at pH 7. Cells were harvested at the cell density of 5×10^6^ and then subjected to immunoblot analyses with a mixture of antibodies against RpoS, OspC, or FlaB (internal control). The bands corresponding to each protein are indicated on the right. (**D**) Overexpression of Pta reduces temperature and cell density-induced activation of the Rrp2-RpoN-RpoS pathway. Wild-type *B. burgdorferi* strain B31 13A (-) or the strain carrying *flaBp-pta* (+) were cultivated either at 23 or 35°C in the standard BSK-H medium. Cells were harvested at the late-logarithmic growth phase (5×10^7^ spirochetes/ml) and then subjected to immunoblot analyses. (**E**) *In vitro* phosphorylation of recombinant Rrp2 by acetyl∼P. Different quantities of purified recombinant Rrp2 or various versions of Rrp2-N were incubated with [^32^P]acetyl phosphate and the reactions were terminated at 15 or 30 min. The reaction mixtures were separated by SDS-PAGE followed by exposure on Kodak X-ray film.

Acetyl∼P is the intermediate in the acetate kinase (Ack) – phosphate acetyltransferase (Pta) pathway. *B. burgdorferi* possesses genes predicted to encode both Ack (BB0622) and Pta (BB0589) [17] (**Fig. 7A**). However, the *B. burgdorferi* genome encodes neither an AMP-ACS pathway that converts acetate to acetyl-coA nor other known pathways that produce acetyl-CoA. It also lacks the TCA cycle which utilizes acetyl-CoA for energy production. The genome does have a mevalonate pathway (BB0683-BB0688) that requires acetyl-CoA for cell wall synthesis. Therefore, the Ack-Pta pathway appears to be the sole pathway for biosynthesis of acetyl-CoA required for cell wall synthesis

As a short-chain fatty acid, acetate can diffuse into cells under neutral or acidic conditions [32]. Then the enzyme Ack can convert acetate to acetyl∼P, which in turn is converted to acetyl-CoA by the enzyme Pta. Thus, increasing concentrations of exogenous acetate can elevate intracellular levels of acetyl∼P [32]. To assess whether acetyl∼P plays a role in Rrp2 activation, wild-type *B. burgdorferi* B31-A3 were cultivated in BSK-H medium supplemented with increasing concentrations of sodium acetate (NaOAc) with the final medium pH adjusted to 7.0. In order to detect the effect of acetate, cells were harvested at low density (5×10^6^ spirochetes/ml) when activation of the Rrp2-RpoN-RpoS pathway (monitored by RpoS and OspC expression) is low [37], [38]. As shown in **Fig. 7B**, supplementation of NaOAc to the growth media dramatically increased the expression of OspC and RpoS in a dose-dependent fashion. This increase was not due to an elevated salt concentration (or to osmotic shock) since supplementation of the medium with as much as 150 mM NaCl did not reproduce this effect (data not shown).

### Overexpression of Pta inhibits the activation of the Rrp2-RpoN-RpoS pathway

To determine whether acetate-induced RpoS and OspC expression occurs via the Ack-Pta pathway, we attempted to generate *ack* and *pta* mutants but were unsuccessful. We reasoned that the Ack-Pta pathway may be indispensable for borrelial growth (see discussion). As an alternative approach, we overexpressed Pta in wild-type spirochetes. We reasoned that if acetate-induced Rrp2 activation results from accumulation of acetyl∼P, then overexpression of Pta would reduce the level of acetyl∼P and abolish the acetate effect. A shuttle vector carrying the *pta* gene under the control of the *flaB* promoter was introduced into strain B31 13A. The resulting transformants were cultivated in the presence of 15 mM NaOAc at pH 7.0 and harvested at low cell density (5×10^6^ spirochetes/ml). As shown in **Fig. 7C**, overexpression of Pta dramatically reduced acetate-induced Rrp2 activation as assessed by expression of OspC. These results are consistent with the hypothesis that acetate activates Rrp2 via accumulation of acetyl∼P.

A combination of elevated culture temperature and increased cell density or lowered pH (pH 6.8–7.0) induces RpoS and OspC expression [5], [37], [38], [45], yet the underlying mechanism remains unclear. Since temperature, cell density, and pH are capable of influencing intracellular level of acetyl∼P in other organisms, such as *E. coli* [32], we sought to determine if overexpression of Pta also affects temperature and cell density-induced Rrp2 activation. Thus, spirochetes were cultivated at 23 or 35°C in standard BSK-H and harvested during late exponential growth (∼5×10^7^ spirochetes/ml). Consistent with previous observation, elevated temperature and cell density induced OspC expression in wild-type spirochetes (**Fig. 7D**, the left panel). However, overexpression of Pta dramatically inhibited such effect (**Fig. 7D**, the right panel). These results suggest that the effect of environmental cues such as temperature- and cell density on RpoS and OspC expression might be through the small molecule acetyl∼P.

To determine whether overexpression of Pta would affect mammalian infection by *B. burgdorferi*, we re-constructed a Pta-overexpressing strain in the infectious strain B31-A3. One of the transformed clones harboring *flaBp-pta* had an endogenous plasmid profile identical to that of B31-A3, and was chosen for subsequent infection study. As shown in **Table 3**, overexpression of Pta resulted in a moderate reduction of infectivity; half of the mice (4 out of 8) were infected at the dose of 1×10^3^ spirochetes. This result suggests that the AckA-Pta pathway contributes to mammalian infection, likely by synthesizing acetyl∼P, which can donate its phorphoryl group to Rrp2.

### Acetyl∼P phosphorylates Rrp2 *in vitro*

To determine whether Rrp2 can be directly phosphorylated by acetyl∼P, we performed an *in vitro* phosphorylation assay. Different amounts of purified recombinant Rrp2, Rrp2-N, Rrp2-N(D52A), or Rrp2-N(D52E) were incubated with ^32^P-labeled acetyl∼P in the reaction buffer at 37°C for 15 or 30 min. As shown in **Fig. 7E**, phosphorylated Rrp2 was readily detected in a time- and dose-dependent manner. Furthermore, phosphorylation of Rrp2 requires D52, since wild-type Rrp2-N, but not Rrp2-N(D52A) or Rrp2-N(D52E) could be phosphorylated by acetyl∼P (**Fig. 7E**). These results indicate that acetyl∼P can directly donate its phosphoryl group to Rrp2 in a histidine kinase-independent manner.

## Discussion

The discovery of the central regulatory network, the Rrp2-RpoN-RpoS pathway, was a significant advance in *B. burgdorferi* gene regulation. However, the dearth of knowledge regarding the mechanism underlying the activation of this pathway has been a major gap in our understanding of *Borrelia* host adaptation. In this study, we showed that temperature- and cell density-induced Rrp2-RpoN-RpoS activation occurs via a histidine kinase-independent mechanism. We further provided evidence suggesting the hypothesis that the high-energy metabolic intermediate acetyl∼P plays a key role in Rrp2 phosphorylation and, consequently, the activation of the Rrp2-RpoN-RpoS pathway.

In this study we first extended the recent finding by Burtnick *et al.* [6] that Hk2 was not essential for Rrp2 activation under *in vitro* cultivation conditions, by further showing that the *hk2* mutant was capable of activating the Rrp2-RpoN-RpoS pathway in a mammalian host-adapted model and establishing infection in mice. The fact that the *hk2* mutant remained capable of upregulation of OspC and downregulation of OspA in the DMC model (**Fig. 2**) indicates that this sensor kinase and its PAS sensing domain does not play a major in sensing mammalian host-specific signals for RpoS activation. We next tested the hypothesis that Hk1, the only other *B. burgdorferi* histidine kinase with no assigned function, could be responsible for activation of the Rrp2 pathway. We found that the *hk1* and *hk1 hk2* mutants exhibited normal levels of temperature-induced Rrp2-dependent OspC expression. We further found that spirochetes lacking other histidine kinases identified in the *B. burgdorferi* genome, the chemotaxis histidine kinases CheA1 or CheA2, also exhibited normal OspC expression. One caveat is that we have not tested *cheA1 hk2* and *cheA2 hk2* double mutants and thus cannot formally rule out a possible compensatory effect between Hk2 and CheA1 or CheA2.

Several groups have reported the existence of atypical response regulators in other bacteria whose activities do not require phosphorylation of their receiver domains [46]–[48]. These atypical response regulators either do not possess the conserved aspartate residue shown to function as the phosphorylation site (e.g., HP1021 and HP1043 in *Helicobacter pylori*) [46], or lack conserved residues for Mg^++^ chelation, which is essential for phosphorylation (e.g., FrzS in *Myxococcus* or NblR in *Synechococcus*) [47], [48]. However, Rrp2 retains all the conserved residues for phosphorylation (D52), Mg^++^ binding (D8, D9), and signal transduction (T80, F99, K102). Thus, it is unlikely that Rrp2 is an atypical response regulator. Indeed, in this study, we showed that Rrp2 can autophosphorylate using acetyl∼P as its sole phosphoryl donor. Furthermore, overexpression of the phosphorylatable receiver domain of Rrp2 (Rrp2-N), but not variants of Rrp2-N that carry the D52A or D52E mutations, interfered with endogenous Rrp2 activity. This result is consistent with the assumption that Rrp2 activation requires phosphorylation of D52. Another evidence supporting phosphorylation-dependent Rrp2 activation is our previous observation that the ATPase activity of Rrp2, an activity that is essential for its transcriptional activation function, also is dependent on phosphorylation of Rrp2 [15]. Of note, overproduction of a protein from a strong constitutive promoter (e.g., *flaB*) could have pleiotropic effects. An ideal approach to study the function of Rrp2 phosphorylation would be to replace the endogenous copy of *rrp2* with the D52A mutant allele. Despite multiple efforts, however, we failed to generate the desired strain. This lack of success is consistent with previous reports that inactivation of *rrp2* may be lethal [6], [18]. We hypothesize that phosphorylated Rrp2 may be important for cell growth. Consistent with this hypothesis, overexpression of Rrp2 exhibited a moderate growth defect (data not shown).

The finding that activation of RpoS and OspC requires phosphorylation of Rrp2 but does not require any of the four histidine kinases led us to hypothesize that the phosphoryl donor might be a high-energy central metabolic intermediate [29], [31], [32]. Indeed, bioinformatic analysis of the *B. burgdorferi* genome revealed one pathway capable of producing carbamoyl-P (ArcA-ArcB) and one pathway that can synthesize acetyl∼P (Ack-Pta). Loss of ArcA, which should result in the inability to synthesize carbamoyl-P, had no effect upon Rrp2-dependent expression, suggesting that carbamoyl-P does not serve as the phosphoryl donor to Rrp2.

Acetyl∼P is the intermediate of the Ack-Pta pathway. The Ack-Pta pathway functions in acetogenesis through the conversion of acetyl-CoA obtained from pyruvate into acetate; operation of this pathway in the opposite direction enables other bacteria to use acetate as a carbon source by activating acetate to acetyl-CoA, which subsequently enters the tricarboxylic acid (TCA) cycle. In some organisms, such as *E. coli*, the pathway is reversible and thus can function in both acetogenesis and acetate activation [32]. The relatively small genome of *B. burgdorferi*, an obligate parasite, does not encode any enzyme known to convert pyruvate to acetyl-CoA, nor does it encode the enzymes of the TCA cycle. Instead, *B. burgdorferi* performs lactogenesis, converting pyruvate to lactate [17] (Xu H. and Yang, X.F., unpublished result). As such, the main function of the Ack-Pta pathway of *B. burgdorferi* is likely not for converting acetyl-CoA to acetate, but for generating acetyl-CoA from acetate. This acetyl-CoA could then be used for cell wall synthesis (*via* the mevalonate pathway [BB0683-BB0688]) and possibly for other metabolic pathways (**Fig. 7A**). Furthermore, *B. burgdorferi* seems to lack other acetyl-CoA synthetic pathways (e.g., the AMP-ACS pathway, β-oxidation of fatty acids, and several amino acid degradation pathways). Thus, the Ack-Pta pathway appears to be the sole pathway for biosynthesis of acetyl-CoA. If so, one would predict that the Ack-Pta pathway is essential for spirochetal growth. This notion is consistent with the fact that we failed to generate either an *ack* or a *pta* mutant by either targeted mutagenesis or random transposon mutagenesis (data not shown). What's the source of acetate for *B. burgdorferi*? Our measurement showed that acetate concentration in mouse blood and the midgut of fed ticks is ∼1.0 M and ∼1.8 mM, respectively (Xu H. and Yang, XF, unpublished data). One of the ingredients of the BSK-H medium, CMRL, also contains 0.61 mM acetate (other ingredients of this complex medium, such as rabbit serum, also may contribute to the overall levels of acetate). Through diffusion or an unknown transport system, *B. burgdorferi* may obtain sufficient acetate from these environments for acetyl-CoA production.

Acetyl∼P has drawn attention as a global regulator of gene expression via its ability to donate its phosphoryl group to a subset of response regulators under certain environmental conditions [32]. In *E. coli*, the intracellular acetyl∼P concentration can reach levels sufficient to phosphorylate a subset of response regulators [49] and thus influence the biological processes controlled by those proteins [32]. Although we have not yet measured the intracellular acetyl∼P levels to determine if this is also the case in *B. burgdorferi*, we were able to provide three lines of evidence to support the conclusion that acetyl∼P plays an important role in Rrp2 activation: (i) the activation of the Rrp2-RpoN-RpoS pathway can be induced by increasing concentration of exogenous acetate (**Fig. 7B**); (ii) overexpression of Pta reduced acetate-induced activation of the Rrp2-RpoN-RpoS pathway (**Fig. 7C**); and (iii) acetyl∼P served as a phosphoryl donor to Rrp2 *in vitro* (**Fig. 7E**). Note that overexpression of Pta did not completely abolish OspC production, suggesting that a low level of Rrp2 activation still occurs. This might be due to the presence of low levels of acetyl∼P, as overexpression of Pta does not abolish the production of acetyl∼P. Alternatively, Hk2 may contribute to Rrp2 activation. We are currently in the process of testing this possibility by overexpressing Pta in the *hk2* mutant. Nevertheless, this partial inhibition of RpoS and OspC expression by overexpression of Pta is consistent with the *in vivo* phenotype that overexpression of Pta resulted in a moderate reduction of spirochetal infectivity in mice (**Table 3**).

It is well established that the Rrp2 pathway can be activated by many environmental cues such as temperature, pH, cell density, oxygen, and CO_2_ levels [37]–[39], [45], [50], [51]. However, the underlying mechanism for these phenomena has not been elucidated. In this regard, it is striking that virtually all the environmental cues that activate the Rrp2 pathway also have been shown to influence the acetyl∼P pool in *E. coli* [32]. This observation is consistent with our hypothesis that acetyl∼P serves as a signaling molecule that responds to environmental cues and in response activates the Rrp2 pathway. Indeed, we showed that overexpression of *pta* greatly inhibited both temperature- and cell density-induced activation of Rrp2 (**Fig. 7D**), suggesting that elevated temperature and increased cell density activate the Rrp2-RpoN-RpoS pathway in an acetyl∼P-dependent manner. Elevated temperature may increase acetyl∼P levels by enhancing diffusion of acetate into the cells and/or from increased transport efficiency via an unidentified transport system for acetate. Elevated temperature also increases cell growth rates that likely lead to increased levels of acetyl∼P [32], [52]. The effect of increased cell density on acetyl∼P levels, on the other hand, can result simply by a change in extracellular pH. As cell density increases, the culture pH diminishes from 7.5 to 7.0 or lower [38], which favors the passive diffusion of acetate into the cells [32].

One caveat of this study is that we used expression of RpoS and OspC as the readout for Rrp2 phosphorylation. An ideal approach for such study would be directly to detect the phosphorylated form of Rrp2. Unfortunately this approach is not technically feasible since most forms of the Asp-phosphorylation are unstable and there is no antibody available for detecting Asp-phosphorylation. Thus, a common approach for studying phosphorylation of response regulators is to monitor the output product as a result of phosphorylation of a response regulator. In the case of Rrp2, the only direct target gene identified thus far is *rpoS* and therefore, expression of *rpoS* faithfully reflects the activation of Rrp2 modulated by phosphorylation. One concern for this approach is whether the effect on RpoS expression observed in this study is through another transcriptional activator, BB647 (BosR). BB647 is a fur homologue and was recently shown that inactivation of this gene significantly reduced *rpoS* and *ospC* expression [53]–[56]. Although it remains unclear how BosR fits into the Rrp2-RpoN-RpoS pathway, we found that neither overexpression of Rrp2-N nor overexpression of Pta affected the level of BosR (data not shown), suggesting that the effects of Rrp2-N or Pta overexpression on RpoS and OspC was not through BosR, rather through Rrp2.

In summary, we have shown that temperature- and cell density-induced the activation of the Rrp2-RpoN-RpoS pathway proceeds independently of histidine kinases and carbamoyl-P. In contrast, biochemical and genetic manipulation of the acetyl∼P-producing Ack-Pta pathway dramatically impacts activation of the Rrp2-RpoN-RpoS pathway, providing strong evidence that acetyl∼P plays an important role in Rrp2 activation under *in vitro* growth conditions. We also provide evidence showing that, during mammalian infection, the Rrp2-RpoN-RpoS pathway is also activated via an Hk2-independent mechanism and that acetyl∼P plays an important role in this process. Then, what is the function of Hk2? One possibility is that Hk2 may play a role in sensing host signals and activating Rrp2 during the process of tick feeding. In this regard, we have examined the phenotype of the *hk2* mutant in ticks and found that the *hk2* mutant indeed has reduced infectivity via the route of tick infestation. Unfortunately, we have not been able to construct an infectious complemented strain and, thus, have been unable to show restoration of this defect, which prevents us from drawing a definitive conclusion on Hk2 function in the enzootic cycle of *B. burgdorferi*. Nevertheless, this preliminary finding suggests that Hk2 may contribute to Rrp2 activation during the process of tick feeding. In addition, spirochetes likely have increased levels of intracellular acetyl∼P in feeding ticks, as they encounter increased temperature [39], as well as a massive influx of nutrients that leads to a dramatic increase of growth rates during this process [57], [58]. Thus, we postulate that while acetyl∼P plays an important in activating the Rrp2-RpoN-RpoS pathway during mammalian infection, both acetyl∼P and Hk2 are likely involved in integrating complex environmental and host signals to modulate the Rrp2-RpoN-RpoS pathway during the process of spirochetal transmission from ticks to mammals.

## Materials and Methods

### Ethics statement

All animal experimentation was conducted following the NIH guidelines for housing and care of laboratory animals and performed in accordance with Indiana University Institutional regulation after review and approval by the institutional Animal Care and Use Committee at Indiana University.

### Bacterial strains and plasmids

Low–passage, virulent *B. burgdorferi* strain B31-A3 was kindly provided by Dr. P. Rosa (Rocky Mountain Laboratories, National Institute of Allergy and Infectious Diseases, National Institutes of Health) [35]. Strain B31 13A that lacks lp25 was kindly provided by Dr. F. T. Liang (Louisiana State University) [59]. The *rrp2* mutant was described previously [9] [20]. The *cheA1* and *cheA2* mutants were kindly provided by Dr. Li (New York medical college, NY) [44]. Borreliae were cultivated in Barbour-Stoenner-Kelly (BSK-H) medium (Sigma, St. Louis, MO) supplemented with 6% normal rabbit serum (Pel Freez Biologicals, Rogers, AR) at 35°C unless indicated otherwise. A shuttle vector pBSV2 (a gift from Dr. P. Rosa) was maintained in *E. coli* strain TOP10. Relevant antibiotics were added to the cultures in the following final concentrations: 300 µg/ml for kanamycin and 50 ng/ml for erythromycin.

### Construction of the *hk2* mutant

To generate an *hk2* mutant in strain B31-A3, a 2.5 kb fragment containing *hk2* and its surrounding region was amplified with primers hk2-delF and hk2-delR (**Supplemental** **Table S1**) and cloned into the cloning vector pCR-XL-TOPO (Invitrogen). The plasmid was digested with *Hin*d III (19 bp downstream of the 5' end of *hk2*) and *Cla*I (637 bp upstream of the 3' end of *hk2*), and a kanamycin-resistance cassette driven by the *fla*B promoter was then inserted into the disrupted *hk2* gene (**Fig. 1A**). The suicide vector was confirmed by sequencing, and the plasmid DNA was transformed into *B. burgdorferi* strain B31-A3 as previously described [9], [60]. Whole cell lysates from positive clones were analyzed by PCR and Western immunoblot analysis using a monoclonal antibody against Hk2 to confirm marker insertion and inactivation of *hk2*. The plasmid profiles of the *hk2* mutant clones were determined by PCR analyses with twenty-one pairs of primers specific for each of the endogenous plasmids [61]–[63]. Two of the three randomly picked clones had plasmid profiles that were identical to the parental strain B31-A3 [35], and one of these was chosen for further study.

### Cultivation of *B. burgdorferi* B31 within dialysis membrane chambers (DMCs)

Dialysis membrane chambers (DMCs) containing 1×10^3^ organisms diluted from a mid-logarithmic growth culture at 33°C *in vitro*, were implanted into the peritoneal cavities of female Sprague-Dawley rats as previously described [40], [42]. The DMCs were explanted 192 h after implantation; the spirochetes then were harvested, washed with 1x PBS buffer, and then examined by SDS-PAGE and silver staining.

### Construction of the *hk1* mutant and the *hk1 hk2* double mutant strain

To construct a suicide vector for inactivation of *hk1*, regions of DNA corresponding to 1.3 kb upstream and 1.3 kb downstream of *hk1* regions were PCR amplified from B31-A3 genomic DNA. The resulting DNA fragments were then cloned upstream and downstream of an erythromycin-resistant marker (*erm^R^*) within the pCR-XL-TOPO cloning vector, resulting in suicide vector pXY245. The inserts of pXY245 were confirmed by sequencing. The plasmid DNA was transformed into *B. burgdorferi* 297 strain BbAH130 as previously described [9], [60], resulting in a mutant with 3.4 kb deletion within *hk1* (except the 460 bp to the 5' end and 385 bp to the 3' end of *hk1*) and an insertion of the *erm^R^* marker. Loss of *hk1* expression was confirmed by RT-PCR analysis.

To construct the *hk1 hk2* double mutant, the suicide vector pHX-hk2-kan DNA was transformed into the *hk1* mutant. Kanamycin and erythromycin-resistant clones were selected and the loss of *hk2* was confirmed by Western immunoblot analysis using an anti-Hk2 monoclonal antibody.

### Construction of shuttle vectors for overexpression of wild type and mutant Rrp2 N-terminal domains and for overexpression of Pta

To constitutively express the wild-type Rrp2 N-terminal domain, the DNA fragment corresponding to the Rrp2-N terminal region was PCR-amplified from *B. burgdorferi* B31-A3 genomic DNA using primers rrp2-N-F and rrp2-N-R (**Supplemental** **Table S1**). Two restriction sites, *Nde*I and *Pst*I, were incorporated into the designated primers and used for insertion of the digested PCR fragment into the pBSV2-derived shuttle vector pJD55 [4] harboring a *flaB* promoter. Thus, expression of Rrp2-N was placed under the control of the *flaB* promoter, *flaBp-Rrp2-N*. The resulting shuttle vector, pJD55/rrp2-N, was verified by sequencing and then transformed into B31 13A and B31-A3.

To introduce a single amino acid substitution (D52A or D52E) into the Rrp2-N terminal domain on pJD55/rrp2-N, site-directed mutagenesis was carried out by using the QuikChange II XL Site-Directed Mutagenesis Kit (Stratagene, La Jolla, CA) with the mutagenic PAGE-purified primers D52A-F/D52A-R and D52E-F/D52E-R (**Supplemental Table S1**) as described by the manufacturer. Briefly, PCR was carried out as follows: 95°C for 50 seconds, 60°C for 50 seconds, 68°C for 10 minutes and 18 cycles. The resulting shuttle vectors with point mutations in Rrp2-N were verified by sequencing and designated pJD55-Rrp2-N(D52A) and pJD55-Rrp2-N(D52E), respectively.

To overexpress Pta, the DNA fragment corresponding to *pta* (*bb0589*) was PCR amplified from *B. burgdorferi* B31-A3 genomic DNA using primers Bb589F and Bb589R (**Supplemental** **Table S1**) and then subsequently cloned into pJD55, which places *pta* under the control of the *flaB* promoter. The resulting shuttle vector was verified by sequencing and then transformed into B31 13A and B31-A3.

### Construction of the *arcA* (*bb0841*) mutant by transposon mutagenesis

The *arcA* mutant was generated by transposon-mediated mutagenesis as part of an on-going transposon signature tagged mutagenesis (STM) study. Briefly, twelve independent mutant libraries, each having a unique 7 bp sequence tag, were created using modified versions of the suicide plasmid pMarGentKan derived from pMarGent [64] (kindly provided by Dr. P. E. Stewart, Rocky Mountain Laboratories, National Institutes of Health, Hamilton, MN). The resulting plasmids were transformed into *B. burgdorferi* B31 5A18; transformants were selected on solid BSK-II media containing 200 µg/ml of kanamycin and 40 µg/ml of gentamicin as described previously [65]. Transposon insertion sites were determined by restriction digestion of the *Borrelia* genomic DNA, plasmid rescue in *E. coli*, and sequencing, as described previously [1].

### Sodium dodecyl sulphate-polyacrylamide gel electrophoresis (SDS-PAGE) and immunoblotting

SDS-PAGE and immunoblot analyses were performed as previously described [66]. Monoclonal antibodies against OspC, RpoS, and FlaB were described previously [20], [38]. Monoclonal antibodies against Rrp2 and HK2 were produced using a previously described method [66]. Rrp2-N fragments were detected using a previously reported polyclonal rat antiserum specific against full length Rrp2 [18].

### Mouse infection via needle inoculation

Three or four week-old C3H/HeN mice (Harlan, Indianapolis, IN) were subcutaneously inoculated with spirochetes at a dose of 10^5^ spirochetes per mouse. Ear punch biopsy and tissue samples (skin, heart, spleen and joint) were collected at the time points indicated for each experiment and cultured in BSK-H medium supplemented with 1× *Borrelia* antibiotic mixture (Sigma, Saint Louis, MO). A single growth-positive culture was used as the criterion for infection of each mouse. All animal protocols were approved by the Institutional Animal Care and Use Committee at Indiana University.

### Quantitative RT-PCR (qRT-PCR)

RNA samples were extracted from *B. burgdorferi* cultures using the RNeasy® mini kit (Qiagen, Valencia, CA) according to the manufacturer's protocols. Three independent culture samples were used for each strain. Digestion of contaminating genomic DNA in the RNA samples was performed using RNase-free DNase I (Promega, Madison, WI), and removal of DNA was confirmed by PCR amplification using primers specific for the *B. burgdorferi flaB* gene [67]. The cDNA was synthesized using the SuperScript III reverse transcriptase with random primers (Invitrogen, Carlsbad, CA). To quantify the transcript levels of *ospC*, an absolute quantitation method was used by creating a standard curve in qPCR assay by following the manufacture's protocol (Strategene, La Jolla, CA). Briefly, a cloning vector containing the *ospC* gene serves as standard template. A series of ten-fold dilution (10^0^ to 10^7^ copies/µl) of the standard template was prepared and qPCR was performed to generate a standard curve by plotting the initial template quantity against the Ct values for the standards. The quantity of the *ospC* and *flaB* in cDNA samples were calculated by comparing their Ct values of the Standard Curve plot. Both standards and samples were performed in triplicate on an ABI 7000 Sequence Detection System using GREEN PCR Master Mix (ABI, Pleasanton, CA). Levels of *ospC* transcript were reported as per 1000 copies of *flaB* transcripts.

### Expression and purification of recombinant Rrp2-N, Rrp2-N/D52A and Rrp2-N/D52E

Purification of recombinant Rrp2 protein was described previously [15]. The PCR fragments encoding Rrp2-N, Rrp2-N/D52A and Rrp2-N/D52E were cloned into the expression vector pGEX4t-2 with a glutathione *S*-transferase (GST) at the N-terminus. Fusion proteins GST-Rrp2, GST/Rrp2-N, GST/Rrp2-N/D52A and GST/Rrp2-N/D52E were expressed in *E. coli* under inducible condition of 1 mM IPTG at 37°C for 6 hours. Proteins were purified from cell lysates using GST SpinTrap (GE Healthcare, Piscataway, NJ) according to the manufacturer's manual.

### *In vitro* phosphorylation assay

[^32^P]acetyl phosphate was synthesized as described by Quon *et al*. [68]. Briefly, the reaction mixture includes 0.3 U *E. coli* acetate kinase (Sigma), 10 µCi of [^32^P]ATP (6000 Ci/mmol, PerkinElmer) in AKP buffer (25 mM Tris-HCl [pH 7.4], 60 mM KOAc, 10 mM MgCl_2_; final pH 7.6) and was incubated at room temperature for 20 min. [^32^P]acetyl phosphate was used either without further treatment or with further purification by filtering through a 30 kDa cut-off membrane to remove acetate kinase (Amicon ultra with 30 kDa cut-off, Millipore). [^32^P]acetyl phosphate was mixed with recombinant Rrp2 (2.5 µl, 0.7 or 1.4 µg), Rrp2-N (2 µg), Rrp2-N/D52A (2 µg), Rrp2-N/D52E (2 µg) for 15 min or 30 min at 37°C. The reaction was terminated by addition of SDS-PAGE loading buffer and then loaded to 12% SDS-PAGE without boiling. The gel was then exposed to a Kodak X-ray film.

## Supporting Information

Table S1 Primers used in this study
(0.04 MB DOC)
