# Correction: Role of Acetyl-Phosphate in Activation of the Rrp2-RpoN-RpoS Pathway in *Borrelia burgdorferi*

**DOI:** 10.1371/annotation/88293342-3534-4b8b-8800-c63a8c86bf6d

**Published:** 2010-12-14

**Authors:** Haijun Xu, Melissa J. Caimano, Tao Lin, Ming He, Justin D. Radolf, Steven J. Norris, Frank Gherardini, Alan J. Wolfe, X. Frank Yang

The seventh author's name was spelled incorrectly. The correct name is: Frank Gherardini. The affiliation for the third author was incorrect. Tao Lin is not affiliated with #4 but with #5 Department of Pathology and Laboratory Medicine, University of Texas Medical School at Houston, Houston, Texas, United States of America. 

