# Supplementary material for: Role of Acetyl-Phosphate in Activation of the Rrp2-RpoN-RpoS Pathway in Borrelia burgdorferi
Source: PLoS Pathog. 2010 Sep 16;6(9):e1001104. doi: 10.1371/journal.ppat.1001104 (PMC2940757; doi:10.1371/journal.ppat.1001104)
Supplement: Table S1 — Primers used in this study (0.04 MB DOC) [file ppat.1001104.s001.doc]

**Supplemental Table 1. Primers used in this study**

| Primer | Sequence^a^ | Purpose |
| --- | --- | --- |
| rrp2-N-F | 5´-catatgttgaaaatgagcaaaatacttg-3´ | amplify *rrp2* N-terminal receive domain |
| rrp2-N-R | 5´-ctgcagaat attagcattttcattatcgt-3´ | same as above |
| D54A-F | 5´-cttgatgtaataataagcgctctgagaatgccccagatatctgg-3´ | PCR primer for *rrp2* N-terminal D54A site-directed mutagenesis |
| D54A-R | 5´-ccagatatctggggcattctcagagcgcttattattacatcaag -3´ | same as above |
| D54E-F | 5´- cttgatgtaataatatctgagctcagaatgccccagatatctgg-3´ | PCR primer for *rrp2* N-terminal D54E site-directed mutagenesis |
| D54E-R | 5´-ccagatatctggggcattctgagctcagatattattacatcaag-3´ | same as above |
| qflaB-F | 5´-accagcatcactttcagggtctca-3´ | qRT-PCR primer for *flaB* |
| qflaB-R | 5´-cagcaatagcttcatcttggtttg-3´ | same as above |
| qOspC-F | 5´-tagcgggagcttatgcaatatcaacc-3´ | qRT-PCR primer for *ospC* |
| qOspC-R | 5´-catcaattttttcctttaatccttca-3´ | same as above |
| hk2-del-F | 5´-ttttcctttttaatttcctctt-3´ | construct the *hk2* suicide vector |
| hk2-del-R | 5´-agcactacttgtattttttttg-3´ | same as above |
| A | 5´-atgaataattttttcaaaaaagc-3´ | verify the *hk2* mutant |
| B | 5´-atgagccatattcaacgggaa-3´ | same as above |
| C  Bb589F  Bb589R | 5´-ttttttcttctctattgt tttcctttg-3´  5´-ctcgagcatatgttgtattctttttataaggt-3´  5´-agatctttaaatgcttatcattaaagcact-3´ | same as above  amplify *bb589*  same as above |

^a^ Restriction enzyme sites are underlined.
